# Supplementary figures and images for: Enteroaggregative E. coli Adherence to Human Heparan Sulfate Proteoglycans Drives Segment and Host Specific Responses to Infection
Source: PLoS Pathog. 2020 Sep 28;16(9):e1008851. doi: 10.1371/journal.ppat.1008851 (PMC7553275; doi:10.1371/journal.ppat.1008851)

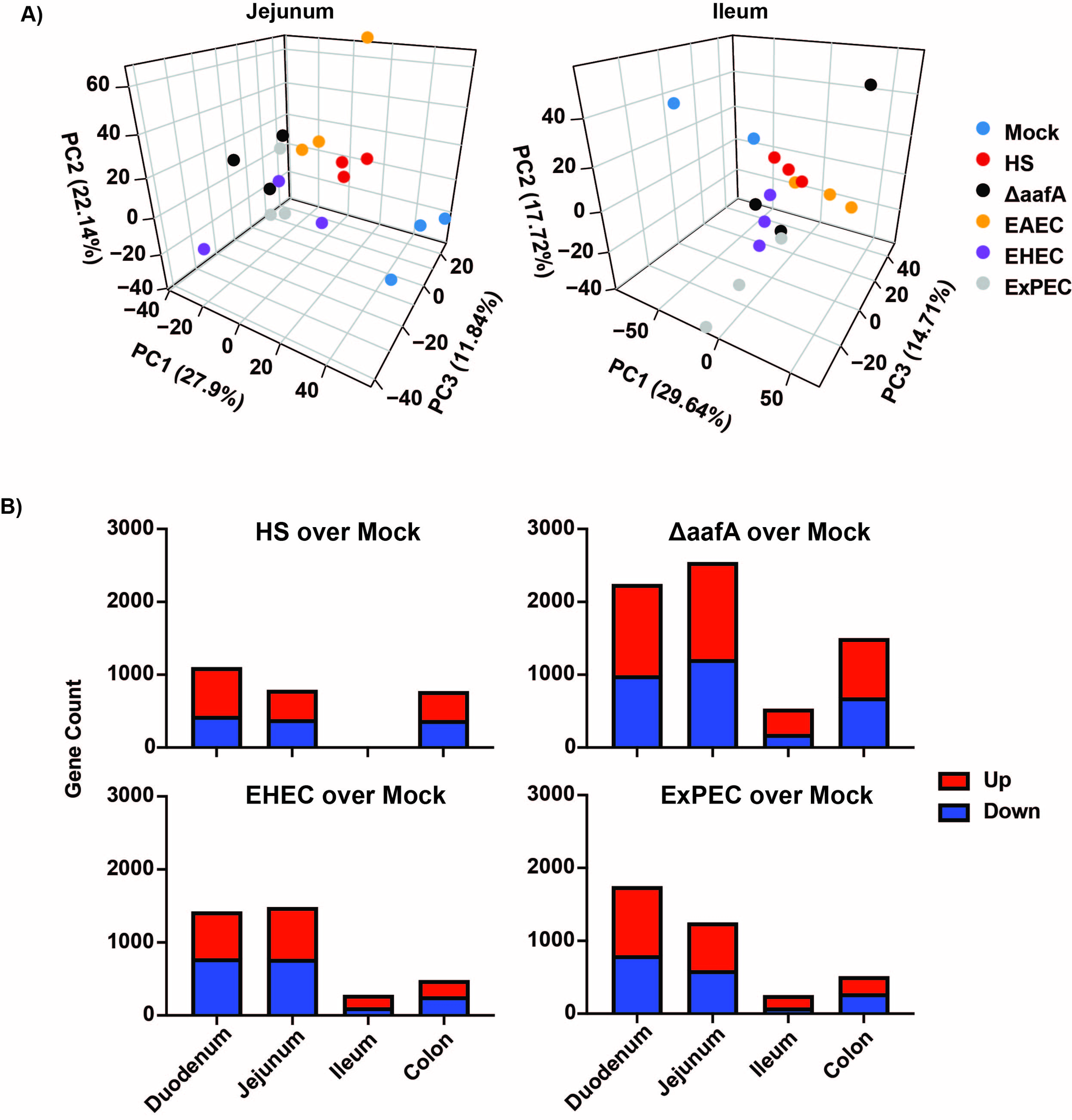

Supplement: S1 Fig — (A) Principal component analysis of jejunum and ileal monolayers demonstrating the variability observed in the samples (infected and mock controls). (B) Total number of significant genes identified for HS, ΔaafA, EHEC and ExPEC-infected groups (BH adjusted p-value < 0.05). (TIF) [file ppat.1008851.s001.tif]

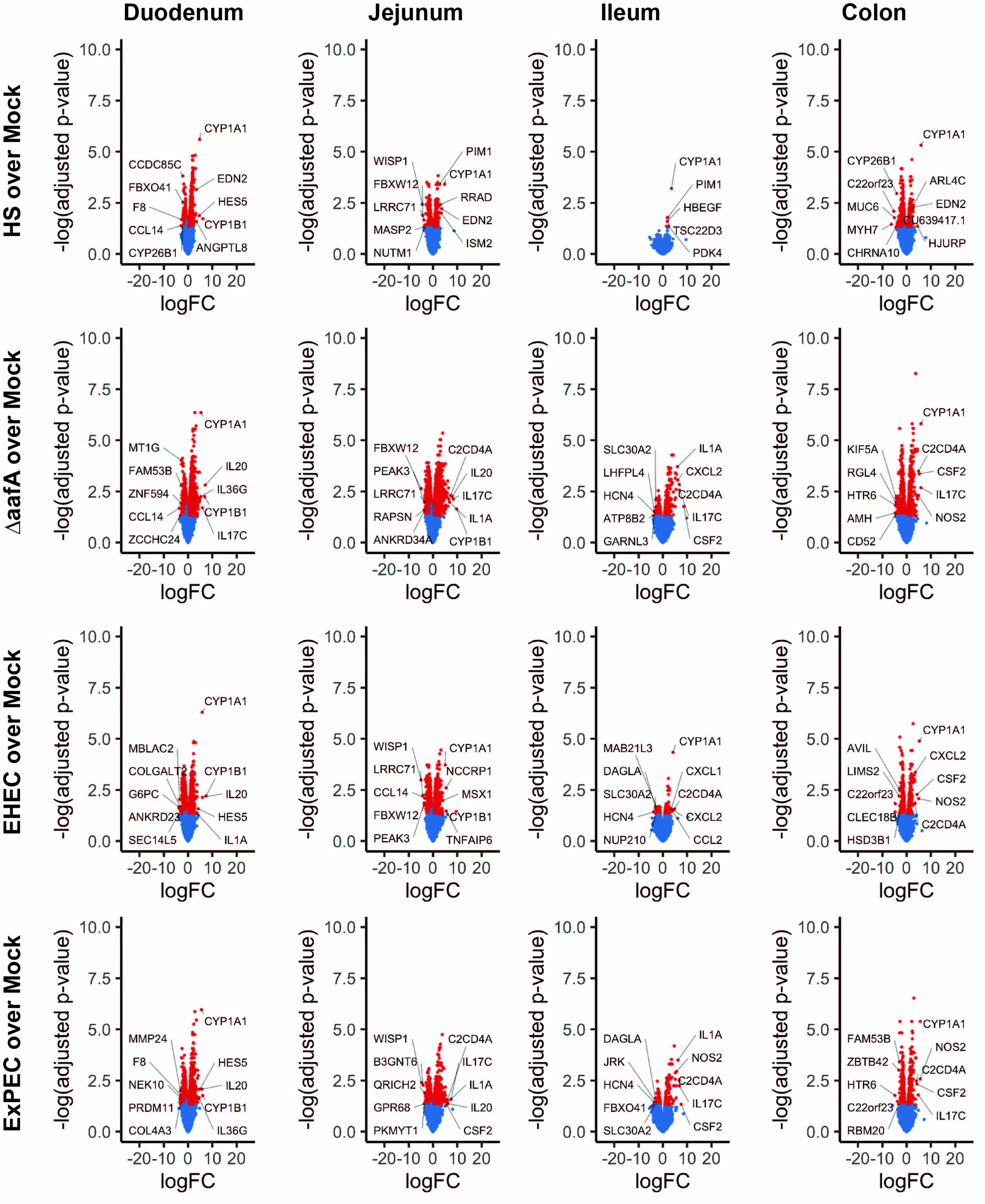

Supplement: S2 Fig — Red dots indicate genes that have an adjusted p-value of at least 0.05 with a linear fold change of at least 1.5. (TIF) [file ppat.1008851.s002.tif]

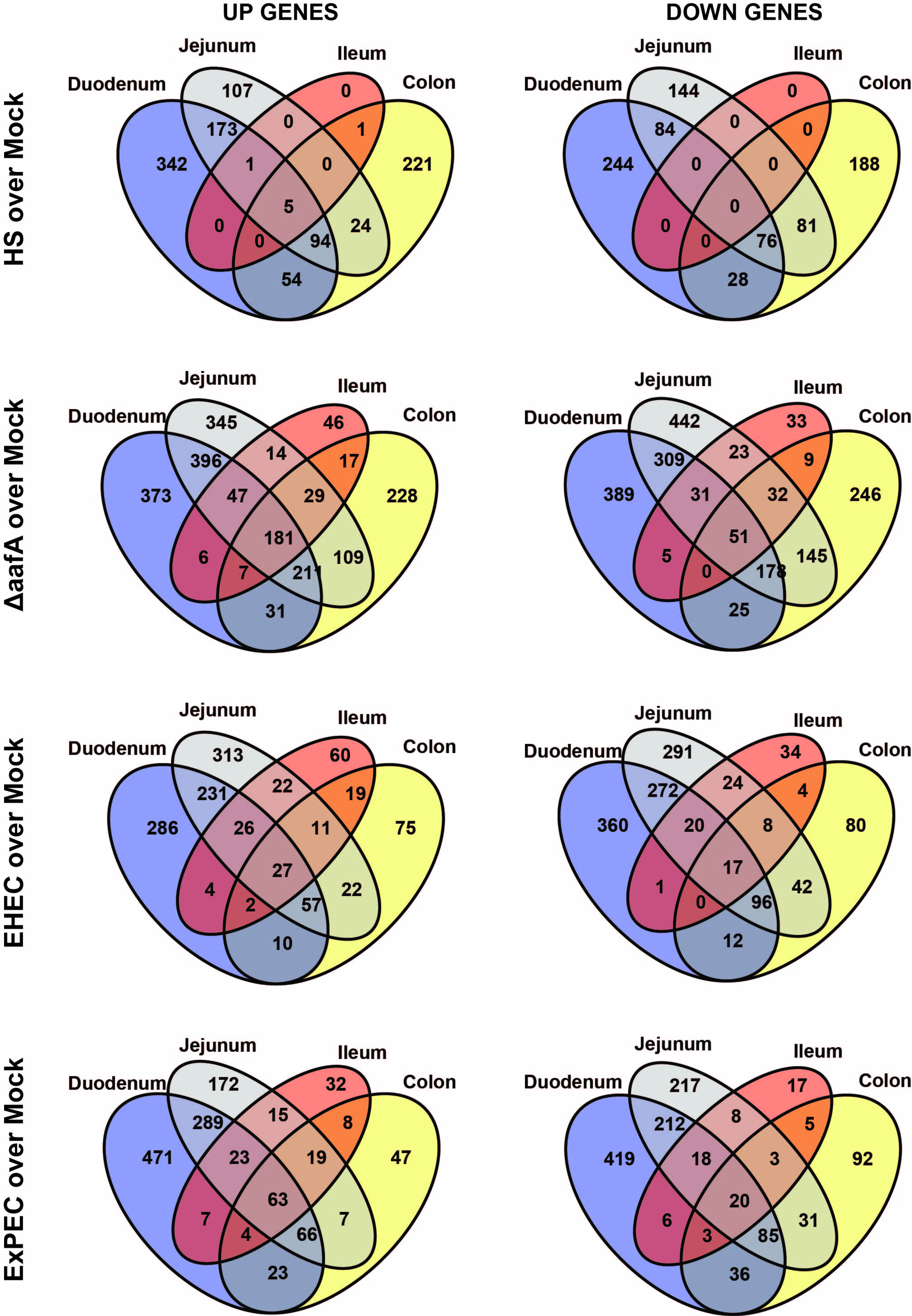

Supplement: S3 Fig — (TIF) [file ppat.1008851.s003.tif]

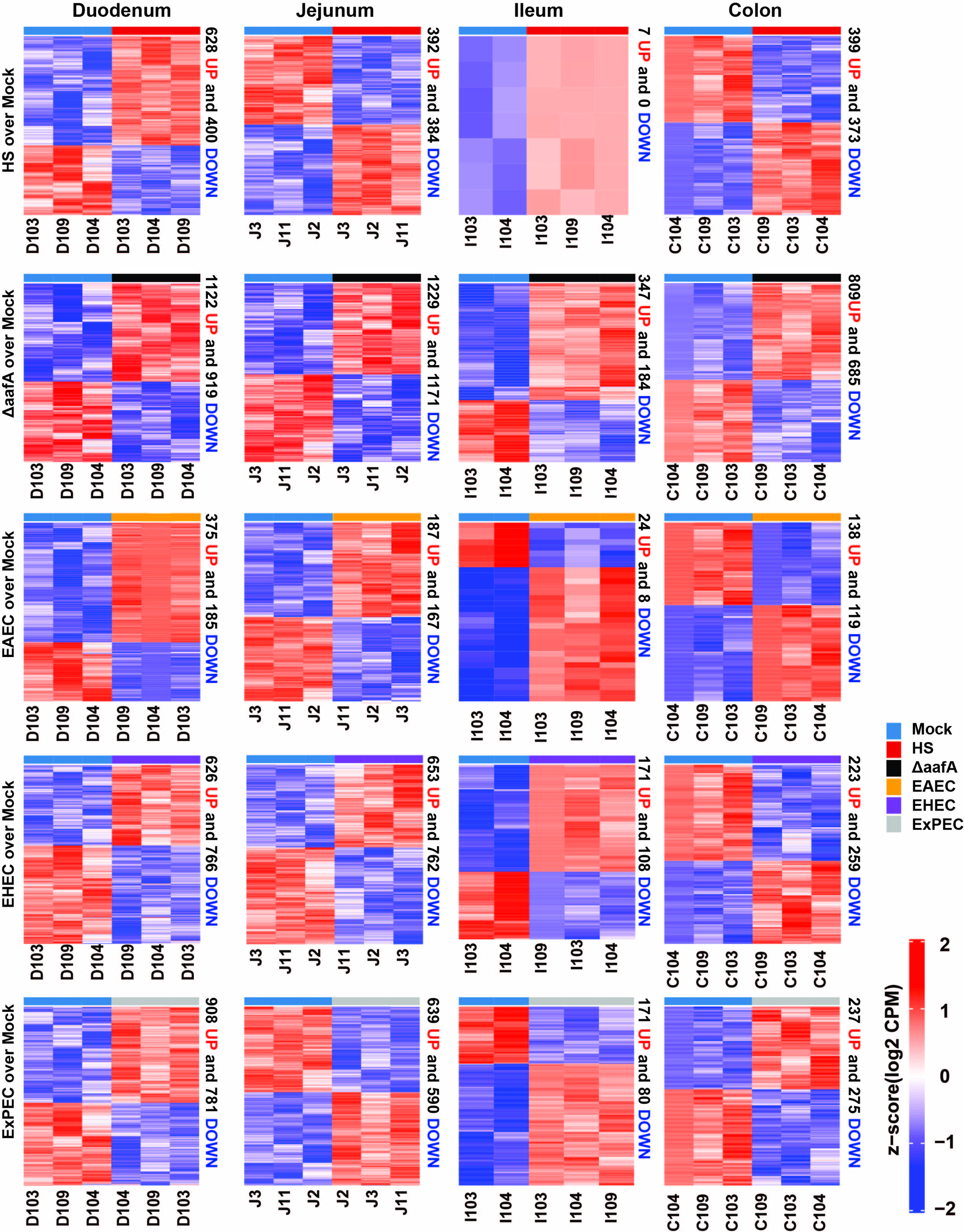

Supplement: S4 Fig — The number of significant genes is indicated on the right of each individual heatmap. (TIF) [file ppat.1008851.s004.tif]

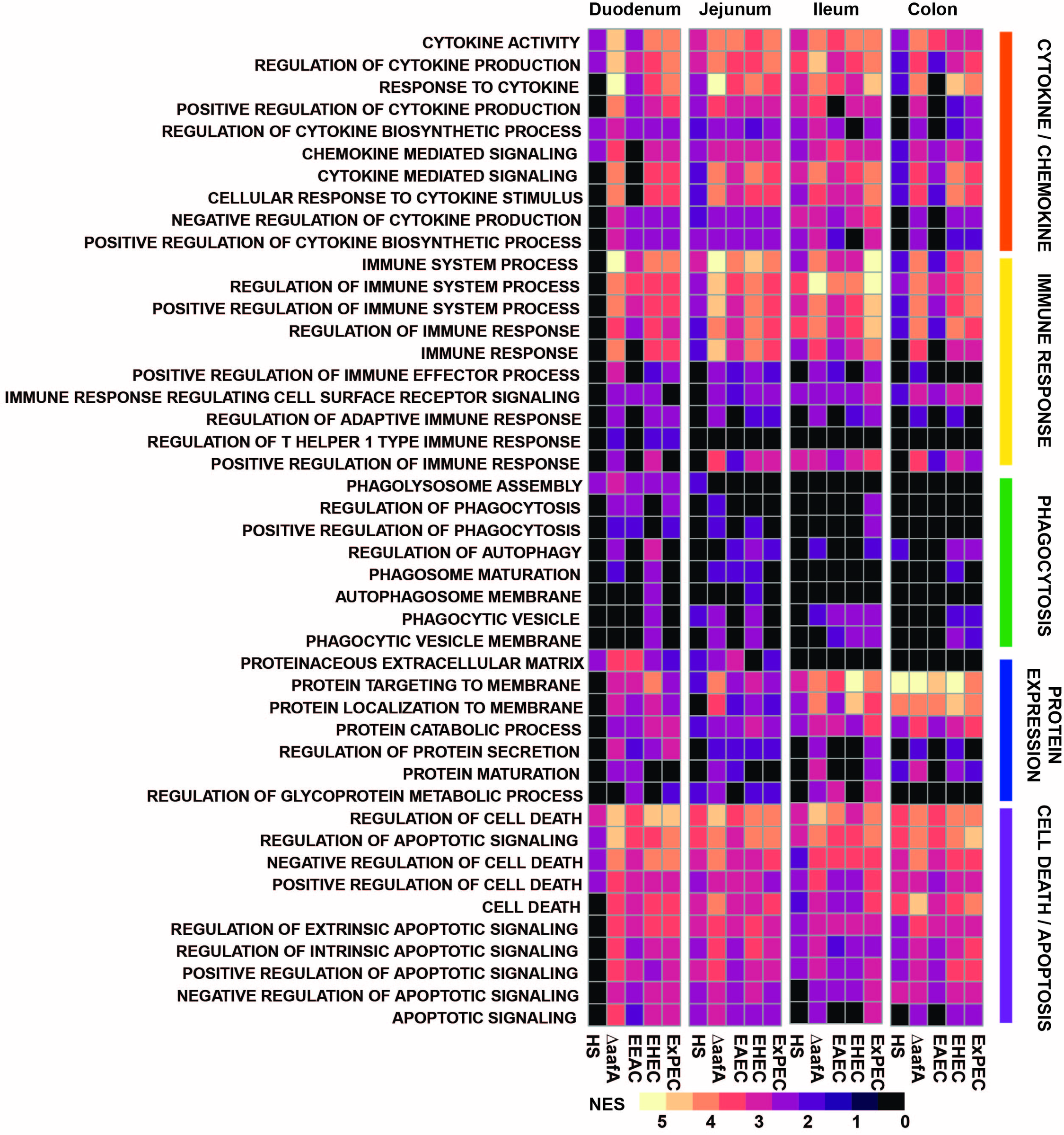

Supplement: S5 Fig — False discovery rate (FDR) of 0.05 was used in the pathway filtering. (TIF) [file ppat.1008851.s005.tif]

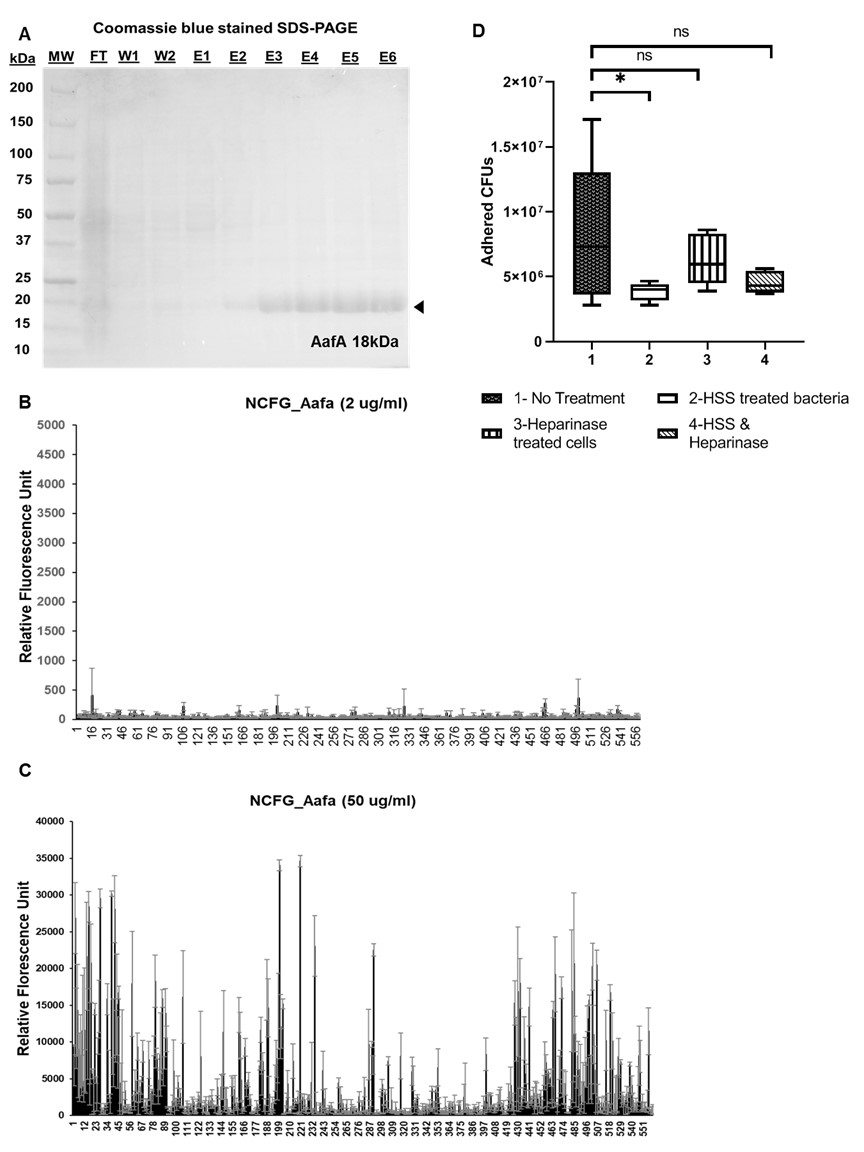

Supplement: S6 Fig — (A) Coomassie blue stained SDS-PAGE of AafA. Molecular weight ladder (MW), Flow through (FT), Washes 1 and 2 (W1-2), and Elutes 1–6 (E1-6) of AafA-His protein at 18kDa after purification by Ni-NTA chromatography. (B-C) Binding pattern of AafA at 2μg/ml and 50μg/ml on a pan glycan array (composed of >500 synthetic glycan) shows preferential binding to charged glycans. (D) Quantification of the total level of EAEC adherence to HIEMs. EAEC or HSS-EAEC were incubated with 2D differentiated colon 109 monolayers that were either mock treated or with Heparinase III and adherence quantified as described in the Methods. (TIF) [file ppat.1008851.s006.tif]

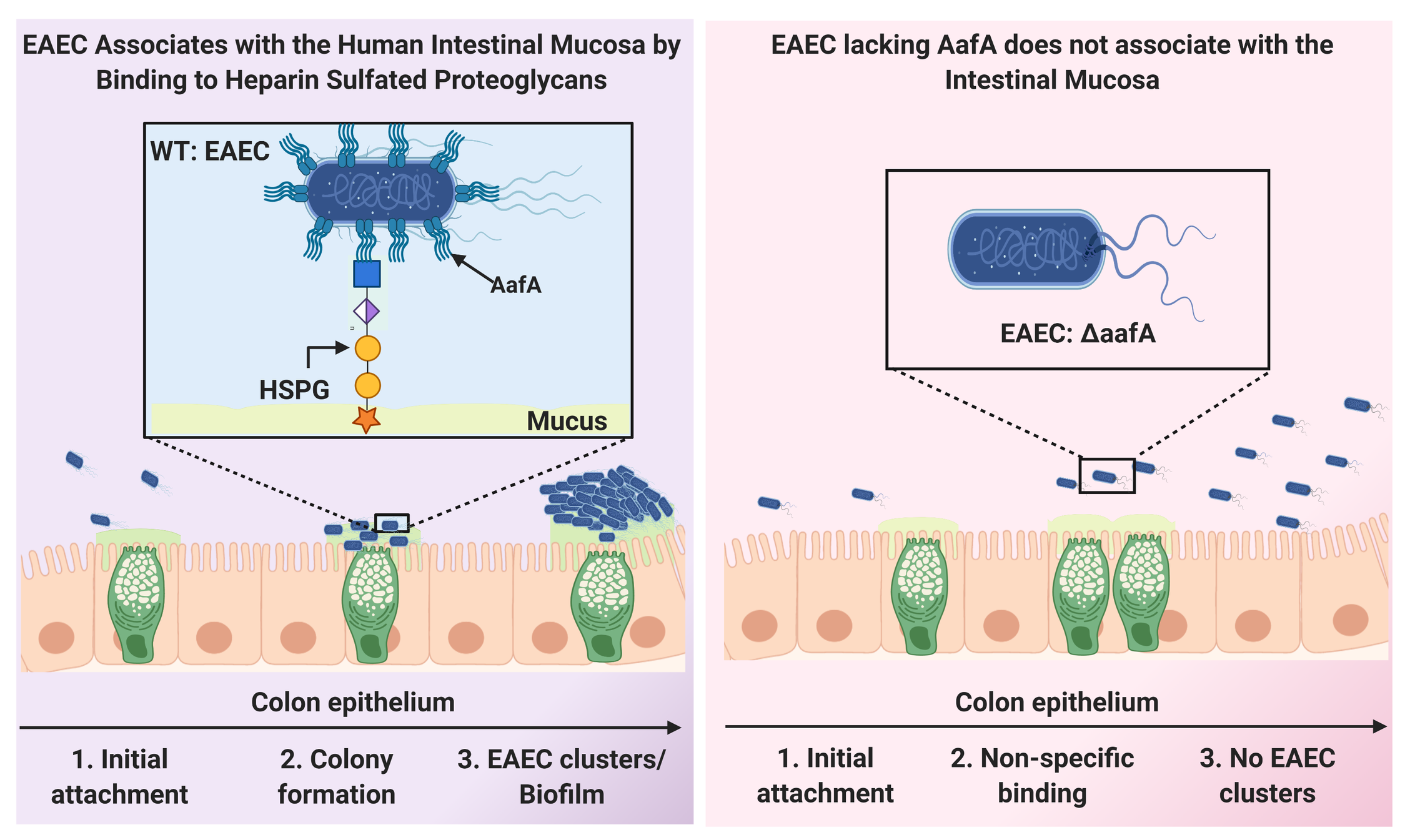

Supplement: S7 Fig — (TIF) [file ppat.1008851.s007.tif]
