# Supplementary material for: Enteroaggregative E. coli Adherence to Human Heparan Sulfate Proteoglycans Drives Segment and Host Specific Responses to Infection
Source: PLoS Pathog. 2020 Sep 28;16(9):e1008851. doi: 10.1371/journal.ppat.1008851 (PMC7553275; doi:10.1371/journal.ppat.1008851)
Supplement: Attachment — Submitted filename: Response to reviewers_Rajan_Maresso_PP.docx [file ppat.1008851.s013.docx]

**Editor’s comments**

Thank you very much for submitting your manuscript "Enteroaggregative E. coli Adherence to Human Heparan Sulfate Proteoglycans Drives Segment and Host Specific Responses to Infection" (PPATHOGENS-D-20-01075) for review by PLOS Pathogens. Your manuscript was fully evaluated at the editorial level and by 3 independent peer reviewers. The reviewers appreciated the attention to an important topic but identified some aspects of the manuscript that should be improved. We therefore ask you to modify the manuscript according to the review recommendations before we can consider your manuscript for acceptance. Your revisions should address the specific points made by each reviewer.

Response: We thank the editorial board and reviewers for the critique of our manuscript. We are encouraged by the overall positive reviews and have responded accordingly with a point by point response.

In particular you should note that all three reviewers felt that the manuscript was of importance, but the first reviewer had some concerns that would require additional experimentation. In particular, this reviewer requested that you consider complementation of the *aafA*-mutant of EAEC strain 042. I agree that to completely fulfill "molecular Koch's postulates" that this complementation is required. In you earlier mBio report, you also did not use a complemented strain. In that report I did not find any reason to why complementation was not attempted. As is true for many strains of E. coli, including clinical isolates, there is sometimes difficulty encountered when trying to perform multiple genetic manipulations as opposed the case with "domesticated" laboratory strains. Has complementation been attempted for this strain? If complementation has been attempted, but not successful, this could be noted. While these data would be useful if available, I am willing to forego this suggestion if you present rationale.

Response: This is an excellent point. It would have been wonderful if we could have complemented this strain. We tried for 18 months to attain this strain using overexpression of the gene from a plasmid, transduction with phage, and recombineering. None of these techniques allowed us to select a clone that expressed full-length *aafA*. Many clones were attained that harbored mutations, but none of which we deemed would be functional. We think there were two problems. First, the parent strain 042 is highly resistant to several antibiotics that are used as selectable elements in the procedures described above. Even when we switched to other antibiotics, such as kanamycin which 042 is sensitive to, we still were not successful. It is possible that this clinical strain rejects foreign DNA and that *aafA* is partially toxic when expressed to above-normal levels. This is the reason why we did not complement in the mentioned mBio paper (as noted by the editor above) and why we left it out of this study as well.

That being said, we do not think the results or interpretation presented either in the mBio paper or this study are impacted by the lack of complementation. Here is why. First, we sequenced the mutant strain and the only change from the wild-type parent is the lack of *aafA*. This means the results we see cannot be due to polar effects at some other loci of the genome Second, we used an entirely independent experiment to essentially confirm the mutant results. This was achieved by the purification of AafA and identification of HSPGs as its ligand. When the wild-type strain was added to HIEs in which HSPGs were either enzymatically removed or via competition with soluble heparin, there was little to no binding by the wild-type. This essentially mirrors the results with the *aafA* mutant strain.

It should be noted, as a last result, we did reach out to a colleague to attain the complemented strain, but, and understandably so, this investigator was a bit concerned releasing these constructs to our group due to perceived conflicts of our NAMSED team with a PPG recently attained by this colleague. This left us with no real options to attain this complemented strain.

We would like to take the Editor up on the generous offer to forgo this experiment. It’s been a nightmare (for whatever reason) but we think the totality of the study, along with the points indicated above, demonstrate that concerns over complementation are not justified.

(As a side note, I would ask that you check your references). For example in the Material and Methods, reference to the EAEC strain 042 is reference "99". This paper does not refer to this strain by my reading but is instead a paper concerning EAF in EPEC strain E2348/69. Similarly in the text [line 129] you refer to EHEC strain 86 [which I believe is more completely referred to as "8624" via reference 39. This reference does not use 8624, but does use EDL933 as a "prototypic" EHEC strain. I did not exhaustively go through your references, but since I found these two when I looked, I would ask that you carefully look through the entire reference set. You should note that reviewer 3 also had some questions about the sources of your strains.

Response: Thank you. We are not sure what happened, perhaps a mix up since many of the authors edited this document and there could have been some formatting issues. All the references have been updated. We apologize for the inconvenience.

Line 127-131

Line 573-574

Another area of specific concern raised by both reviewers 1 and 2 concerns the cloning and expression of *aafA*. My read is that you expressed the entire protein, without the leader peptide. Clarification would be helpful and also addressing the need for the linker region you added. Of course, a point-by-point of the full set of concerns from the 3 reviewers will be required, but I wanted to highlight what I felt were major issues, especially regarding the need for additional experimentation raised by the first reviewer.

Answer: Thank you, this is a good point. We could have been clearer.

AafA is the major subunit of the AAF/II pilus which is assembled by the chaperone/usher pathway. Pilus polymerization in the periplasmic space occurs by donor-strand exchange which involves the flexible N-terminal extension (donor strand/residues 1-10) of the incoming pilin subunit binding to the hydrophobic groove of the adjacent pilin subunit replacing the G strand of the chaperone and complementing the Ig-like fold of the adjacent subunit (Remaut et al. 2006 reference #113). The entire protein is expressed however the residues are rearranged to synthesize a donor strand complemented or self-complementing AafA. Residues 1-10 (donor strand) are linked to the C-terminus to replace the missing G strand which allows the donor strand to bind the hydrophobic groove, therefore creating a self-complementing AafA monomer (Yang et al. 2011 reference #114 and Berry et al. 2014 reference #115). The signal/leader peptide is removed to express and retain AafA in the cytosol so the soluble protein can be purified from the cytoplasmic fraction.

To clarify and provide the information needed to understand this construct, we added Lines (700-726) in the Materials and Methods, now stated as: “AafA is the major subunit of the AAF/II pilus which is assembled by the FGL (long looped) chaperone/usher mechanism (111-112). During pilus polymerization, the flexible N-terminal extension (donor strand) of AafA binds to the hydrophobic groove of the adjacent subunit to complete the Ig-like fold, referred to as donor-strand exchange (113-115). To generate a donor-strand complemented AafA expressed in the cytoplasm, the insert consisted of a 2-residue linker followed by residues 11-135 at the N-terminus, a ten-residue linker, and residues 1-10 at the C-terminus ligated into vector pQE-30 (Qiagen) using restriction sites BamHI and HindIII. The AafA sequence (114) was obtained from (GenBank: AAB82330.1), and the signal peptide was removed to express and retain the protein in the cytoplasm. Constructs with this rearrangement of residues and the incorporated linker allow for conformational flexibility between the N and C-terminus and have been shown to produce soluble and stable donor strand complemented AafA monomers (113-115). To separate the His6 tag and also facilitate the flexibility, a short Gly-Ser linker was also incorporated at the N-terminus (atg cat cac cat cac cat cac ggt agt aat ttt tgt gat ata acg ata aca ccg gct aca aat cgt gat gtc aac gtt gac agg agc gca aat atc gac ctg agt ttt act att aga caa ccg caa cgc tgc gct gat gct ggt atg cga ata aaa gct tgg ggg gaa gcc aat cac ggt caa tta ctg ata aaa cct caa gga gga aat aaa tca gca gga ttc act ctg gcc tct cct agg ttt tct tac att ccg aat aat cca gca aac att atg aat gga ttt gtt ctt acg aat cct ggt gtt tat caa tta gga atg cag ggc tca att aca ccg gct atc ccg cta cga cca ggc cta tat gaa gta gta tta aat gct gag ctt gtg aca aat gat aac aag caa aat gca act gcg gta gca aaa act gcg acc agt act atc act gta gtg taa).”

Reviewer #1

Reviewer #1: Major points:

1. Lane 128 and Fig 1. Strain 86 is an EHEC non-Stx- producing strain, so the authors might want to clarify the reason of using such stx-negative strain for their experiments.

Response. We incorrectly labeled the strain. The strain was mislabeled as 86 instead of 8624. Strain 8624 does produce Stx. The correct strain and references have been updated in the revised manuscript (Line 128).

2. Lanes 161-165, a significant concern regarding the interpretation of the results is the use of an *aafA*-negative, which is expected to have a reduced adherence and therefore, a reduced impact in the total gene expression changes is different intestinal segments. This observation raises multiple questions that require attention in this manuscript: a) does the *aafA* mutant strain uses another mechanism to interact with the host cells? b) a complementation experiment is required to confirm that the effect is directly associated with AafA and no other non-specific mutations.

Response. We apologize for any confusion. The *aafA*-negative strain does not adhere to the cells, this is true (Rajan, mBio, 2018 and data herein). However, it is not true that it has a reduced impact on host gene expression. In fact, the *aafA*-negative strain induced the most gene changes of any *E. coli* strain assessed, and in all four enteroid segments examined (SFig. 1B, SFig. 2). This observation was noted in the original manuscript, lines 160-164. There are two interpretations of this data. First, it may be that the *aafA*-negative strain, being unable to adhere, upregulates the production of toxins or other effectors (possibly the SPATES) compared to wild-type, which in turn induces a significant response from the host. A second interpretation is that adherence actually pacifies the host, i.e. the wild-type strain clandestinely adheres without triggering many gene responses in the HIEs and that the *aafA* mutant strain loses this control via loss of adherence. Both interpretations of the data were noted in the manuscript (lines 386-389).

Regarding point B (complementation), and as noted to the Editor above, we tried for 18 months to attain this strain using overexpression of the gene from a plasmid, transduction with phage, and recombineering. None of these techniques allowed us to select a clone that expressed full-length *aafA*. Many clones were attained that harbored mutations, but none of which we deemed would be functional. We think there were two problems. First, the parent strain 042 is highly resistant to several antibiotics that are used as selectable elements in the procedures described above. Even when we switched to other antibiotics, such as kanamycin which 042 is sensitive to, we still were not successful. Second, it is possible that this clinical strain rejects foreign DNA and that *aafA* is partially toxic when expressed to above-normal levels. This is the reason why we did not complement in the mentioned mBio paper (as noted by the editor above) and why we left it out of this study as well.

That being said, we do not think the results or interpretation presented either in the mBio paper or this study are impacted by the lack of complementation. Here is why. First, we sequenced the mutant strain and the only change from the wild-type parent is the lack of *aafA* This means the results we see cannot be due to polar effects at some other loci of the genome Finally, we used an entirely independent experiment to essentially confirm the mutant results. This was achieved by the purification of AafA and identification of HSPGs as its ligand. When the wild-type strain was added to HIEs in which HSPGs were either enzymatically removed or via competition with soluble heparin, there was little to no binding by the wild-type. This essentially mirrors the results with the *aafA* mutant strain.

3. Lanes 190-192, it is not clear to the reader the reason the authors consider ExPEC the “most pathogenic” pathotype and why is more like the *aafA* mutant strain. The data might suggest then that the *aafA*-mutant strain modifies its tropism but maintains its ability to bind to other intestinal segments, causing further damage mediated by other adhesins? If that is the case, the authors need to confirm this finding or confirm the specificity of AafA tissue interaction.

Response. We agree and removed these statements in the revised manuscript. The original contention was that ExPEC was considered more pathogenic simply because systemic infections with this pathotype can lead to bacteremia and death. It is believed there are tens of thousands such infections every year in the U.S. alone. The statement arose from this perspective. The point of this study was to investigate the host response in different segments to different pathotypes. Since statements about relative pathogenicity muddy the water, we removed the references.

4. Lanes 203-205. As indicated above, the drastic changes observed with the *aafA* mutant strain require a complementation analysis to confirm that this is an AafA-specific phenotype and not an artifact of the mutation, because it is difficult to understand how a reduction in adherence will have such a drastic change in host-responses.

Response. As we stated above, we had too much difficulty deriving the complemented strain. Clinical strains are notorious for such properties. We also pointed out above how the results cannot possibly be due to “artifacts” of the mutation.

We explained the results with the mutant strain in two ways. First, it may be that the *aafA*-negative strain, being unable to adhere, upregulates the production of toxins or other effectors (possibly the SPATES) compared to wild-type, which in turn induces a significant response from host. A second interpretation is that adherence actually pacifies the host, i.e. the wild-type strain clandestinely adheres without triggering many gene responses in the HIEs and that the *aafA* mutant strain loses this control via loss of adherence. Both interpretations of the data were noted in the manuscript (lines 386-391)

5. Lane 244 “…different adherence patterns on enteroids” have not been shown in this manuscript. Is this part of a prior publication? Otherwise an example adherence patterns must be included.

Response. The reviewer is correct. It was a part of the publication that led up to this work. The revised manuscript has been updated (Line 252).

6. Lanes 266-270, it is not clear the rationale behind the selection of MUC2 for the staining in Fig 4, because MUC2 was not one of the mucins differentially expressed upon contact with EAEC. It will make more sense to use a MUC that is associated with EAEC interaction.

Response. The point is well taken. We tried this but antibodies to other mucins and/or lectins are either not specific or not well developed. Antibodies to MUC2 are commonly used and of high quality. It is a marker for mucus in general via Goblet cell secretion and has been associated with other diarrheagenic pathotypes, such as EHEC in colonoids. MUC2 was the best option at this point. The revised manuscript now includes the rationale for using MUC2 (Line 268-270).

7. Lanes 305-306, again, it is not clear the reason to incorporate a 10-residue linker to the segment of the *aafA* gene cloned? Is the gene segment sufficient to express the functional binding domain?

Response. Thank you. We just added what we wrote above here as well to be complete.

AafA is the major subunit of the AAF/II pilus which is assembled by the chaperone/usher pathway. Pilus polymerization in the periplasmic space occurs by donor-strand exchange which involves the flexible N-terminal extension (donor strand/residues 1-10) of the incoming pilin subunit binding to the hydrophobic groove of the adjacent pilin subunit replacing the G strand of the chaperone and complementing the Ig- like fold of the adjacent subunit (Remaut et al. 2006 reference #113). The entire protein is expressed however the residues are rearranged to synthesize a donor strand complemented or self-complementing AafA. Residues 1-10 (donor strand) are linked to the C-terminus to replace the missing G strand which allows the donor strand to bind the hydrophobic groove, therefore creating a self-complementing AafA monomer (Yang et al. 2011 reference #114 and Berry et al. 2014 reference #115). The signal/leader peptide is removed to express and retain AafA in the cytosol so the soluble protein can be purified from the cytoplasmic fraction.

To clarify and provide the information needed to understand this construct, we added Lines (700-726) in the Materials and Methods, now stated as: “AafA is the major subunit of the AAF/II pilus which is assembled by the FGL (long looped) chaperone/usher mechanism (111-112). During pilus polymerization, the flexible N-terminal extension (donor strand) of AafA binds to the hydrophobic groove of the adjacent subunit to complete the Ig-like fold, referred to as donor-strand exchange (113-115). To generate a donor-strand complemented AafA expressed in the cytoplasm, the insert consisted of a 2-residue linker followed by residues 11-135 at the N-terminus, a ten-residue linker, and residues 1-10 at the C-terminus ligated into vector pQE-30 (Qiagen) using restriction sites BamHI and HindIII. The AafA sequence (114) was obtained from (GenBank: AAB82330.1), and the signal peptide was removed to express and retain the protein in the cytoplasm. Constructs with this rearrangement of residues and the incorporated linker allow for conformational flexibility between the N and C-terminus and have been shown to produce soluble and stable donor strand complemented AafA monomers (113-115). To separate the His6 tag and also facilitate the flexibility, a short Gly-Ser linker was also incorporated at the N-terminus (atg cat cac cat cac cat cac ggt agt aat ttt tgt gat ata acg ata aca ccg gct aca aat cgt gat gtc aac gtt gac agg agc gca aat atc gac ctg agt ttt act att aga caa ccg caa cgc tgc gct gat gct ggt atg cga ata aaa gct tgg ggg gaa gcc aat cac ggt caa tta ctg ata aaa cct caa gga gga aat aaa tca gca gga ttc act ctg gcc tct cct agg ttt tct tac att ccg aat aat cca gca aac att atg aat gga ttt gtt ctt acg aat cct ggt gtt tat caa tta gga atg cag ggc tca att aca ccg gct atc ccg cta cga cca ggc cta tat gaa gta gta tta aat gct gag ctt gtg aca aat gat aac aag caa aat gca act gcg gta gca aaa act gcg acc agt act atc act gta gtg taa).”

8. Discussion, this is an extremely lengthy discussion and at least it must be reduced by 50%. For example, lanes 344-355 includes an introduction rather than a discussion, particularly since the organotypic model might not be useful to evaluate a vaccine.

Response. The Discussion has been shortened. The following lines in the PRVIOUS VERSION of MS were deleted.

Lines 342-351; 353 355-357, 360, 407, 415-417, 426, 436, 441, 442, 452, 463-468, 534-550. Technically, this constituted a deletion of 21% of the current discussion. We believe what remains in the revised manuscript is critical information that should be disseminated to the community interested in this line of work.

Reviewer #2: (No Response)

Reviewer #3: None

**Reviewer #1: Minor points:**

Response. We thank the reviewer for taking the time to improve these elements. We made all the suggested changes except for one.

1. Lane 92 “…ecological niche; second, what are…”

The suggested change has been recorded and for reference.

2. Lane 96, do you mean “ex vivo” instead of “ex situ”?

Thank you, it has been updated and for reference.

3. Lane 113 and elsewhere. The protein name is “AafA” and the gene name “*aafA*”

The changes have been made.

4. Lanes 120-123, this is a run-on sentence, please re-structure.

Thank you, the sentence was revised to:

“In addition, we assessed responses to five different *E. coli* pathotypes. Each pathotype either causes a unique disease in the intestine or extra-intestinally.”

5. Figure 2, order of panels, panels D and E should be C and D and then panel C should be E. change in the text.

Thank you for the suggestion. We would prefer to keep the current order because we believe it is a concise, streamlined way to present the data that is also logical and symmetrical.

6. Lanes 254-260 and Figure 3. It is not clear which ones are panels 1-4, do you refer to the rows in panels A-C?

Yes, thank you for the suggestion in terminology. It has been updated accordingly (Lines 254-260 and Figure 3 legend).

7. Lane 289 and Fig 5D, it should read “10 μM”

Changed and thank you

8. References, italicize “*Escherichia coli*”, “*in vitro*“, “E. coli“, “in vivo”, “*Campylobacter jejuni*”, “*Staphylococcus aureus*”.

Reference 33 remove all capital letters. Reference 35 “mBio” instead of “MBio”.

Revised, thank you.

Reviewer #2: 1. In the comparison of the WT and *aafA* mutant, it is shown that the mutant induced a much broader host response than the wild type. Is it possible that the fimbrae may have a role in suppressing host response? Were cytokine or innate immune response genes among those that were upregulated in the presence of the mutant but not the wild type?

Response. This is an astute point. We think there are two possibilities. The first is as the reviewer suggests, namely, that the fimbriae may promote a down-modulation of the host response, what we refer to as a passivating effect. This might promote commensalism, or, in general, colonization without overt detection, sort of a stealth-like mechanism. The second hypothesis is that the loss of the fimbriae (and adherence in general) somehow exacerbates the virulent state of EAEC. Along these lines, one idea is that in the absence of adherence it undergoes a type of stress response that leads to the production of secreted SPATES or other virulence factors that activate the innate host response of intestinal epithelial cells. We did notice that the fimbriae-less strain was more cytotoxic to the host cells, which would explain this finding (Supplementary figure 5, apoptosis pathways). In this regard, the host response seemed to be more directed at the regulation of apoptotic and cell-death signaling for the mutant strain, which would be consistent with this idea. On the topic of immune response: to the reviewer’s point, the fimbriae-minus strain did show about a two-fold increase in some chemokines and cytokines above the wild-type, control strain (Supplementary figure 5).

2. Line 304. …amino acids 1-135 of AafA. Is this the full length protein? If so, it would be clearer to say the full length protein was cloned and put the details in the Materials and Methods. If this is a fragment of the protein, some explanation for the fragment chosen would be useful.

Response. Sorry, this was not clear.

The entire mature protein (residues 1-135) is expressed, but the residues are rearranged to produce a donor strand complemented or self-complementing AafA. Residues 1-10 (donor strand) are linked the C-terminus to replace the missing G strand which now allows the donor strand to bind the hydrophobic groove of the N-terminus. This produces a self-complementing AafA monomer (Yang et al. 2011 reference #114 and Berry et al. 2014 reference #116). To provide the background to understand the construct and synthesized protein the procedure in the Materials and Methods section is revised. Lines (700-726), “AafA is the major subunit of the AAF/II variant pilus which is assembled by the FGL (long looped) chaperone/usher mechanism (111,112). During pilus polymerization, the flexible N-terminal extension (donor strand) of AafA binds to the hydrophobic groove of the adjacent subunit to complete the Ig-like fold, referred to as donor-strand exchange (113). To generate a donor-strand complemented AafA (AafA with the donor strand linked to the C-terminus to form the missing G strand referred to as AafA here) expressed in the cytoplasm, the insert consisted of a 2-residue linker followed by residues 11-135 at the N-terminus, a ten-residue linker, and residues 1-10 at the C-terminus ligated into vector pQE-30 (Qiagen) using restriction sites BamHI and HindIII (prepared by Genewiz, USA). The AafA sequence referenced to make the construct as mentioned in Yang et al. (112) was obtained from (GenBank: AAB82330.1), and the signal peptide was removed to express and retain the protein in the cytoplasm.

Constructs with this rearrangement of residues and the incorporated linker for conformational flexibility between the N and C-terminus have been shown to produce soluble and stable donor strand complemented AafA monomers (113-116). To separate the His6 tag while facilitating flexibility a short Gly-Ser linker at the N-terminus was also incorporated (atg cat cac cat cac cat cac ggt agt aat ttt tgt gat ata acg ata aca ccg gct aca aat cgt gat gtc aac gtt gac agg agc gca aat atc gac ctg agt ttt act att aga caa ccg caa cgc tgc gct gat gct ggt atg cga ata aaa gct tgg ggg gaa gcc aat cac ggt caa tta ctg ata aaa cct caa gga gga aat aaa tca gca gga ttc act ctg gcc tct cct agg ttt tct tac att ccg aat aat cca gca aac att atg aat gga ttt gtt ctt acg aat cct ggt gtt tat caa tta gga atg cag ggc tca att aca ccg gct atc ccg cta cga cca ggc cta tat gaa gta gta tta aat gct gag ctt gtg aca aat gat aac aag caa aat gca act gcg gta gca aaa act gcg acc agt act atc act gta gtg taa).”

This new information is inserted in the Methods, lines 700-726.

3. Fig. 3. Some explanation is needed for what is counted as a cluster. It is not clear from the legend what the bars in Fig. 3D mean. Also, the legend says the yellow arrow indicates EAEC clusters but these look more like individual bacteria in the 4^th^ panel.

Response. Agreed, this needs to be clarified. We refer to the EAEC aggregation as a cluster. In the revised manuscript, we changed the word cluster to aggregation. In our previous manuscript on this topic (mBio, Rajan et al, 2018), we defined several ways to classify these bacterial groupings. In general, if there is more than 20 bacteria in a group, it is called aggregative adherence. The details about the error bars, n and p value have been added.

Line 1087-1098

4. Fig. 4 and Fig. 5 legends. DAPI (nuclei) and GFP (EAEC) are defined but the staining for MUC2 and E-cadherin are not stated.

Response. Thank you, the changes have been made in Lines 1100-1119.

5. Fig. 5 D and E. Is this the number of aggregates per field, per sample?

Response. It is aggregates per field. The details have now been added in figure legend

6. Fig. 6. A bit more detail is needed in the legend for D-F. Defining the acronyms and stating that soluble HS is added as a competitor would make this easier for the reader.

Response. This has been done and updated in the legend, thank you

Reviewer #3: For experimental clarity:

This ground-breaking study is likely to drive many other investigations in the field. As such, it would be useful if the methods were very clear for others to follow. Something akin to the Key Resource Table introduced by CellPress should be adopted by all journals.

Response. Thank you for reviewing our study. We address the critiques below.

1. In general, the figure legends lack detail and should include number of repeats and the statistical analyses used. For example: Figure 2E, 3D, 5D-E. What is N? These look like pairwise comparisons, was Tukey’s multiple comparison really used for the statistical analysis?

Response. We have revised the figure legends with more detail. Pasted below are the complete legends for Figures 2, 3, and 5.

Figure 2: RNA-Sequencing analysis reveals a pathogen-specific differential gene expression signature within different segments of intestine. (A) Hierarchical clustering dendrogram of all gene expression changes using the Pearson correlation coefficient among mock and E. coli infected samples for all four segments of intestine (Duodenum, Ileum, Colon from patients 103, 104 & 109 and jejunum from patient 2, 3 & 11 – see Methods) (B) Samples were hierarchically clustered based on the union of differentially expressed genes across all comparisons (FDR<0.05 and fold change exceeding 1.5x). Genes were converted to 1 for up-regulated genes and -1 for down-regulated genes and 0 if it a genes were not significant in a particular signature. Samples were clustered used the Euclidean distance. (C) Heatmap showing EAEC infection-specific regulation of Muc gene expression in HIEs. (D) PAS/AB staining of HIEMs infected with EAEC shows epithelial cell damage. The left panel shows the mock-infected epithelial cell layer whereas the right panel reveals epithelial thinning and damage caused by EAEC adherence (EAEC clusters denoted by arrows). (E) Quantification of epithelial thinning caused by EAEC infection. Scale bars indicate 10µm. Epithelial thickness was quantified from average of 12 images taken at 100x and with field size of 2560x1920 pixel. Unpaired t-test was carried out, error bars denote SEM and ** indicates significantly different (P<0.01), * indicates significantly different (P<0.05).

Figure 3: SEM and TEM images of EAEC adherence to human intestinal enteroid monolayers. (A-C) shows SEM image of Mock, EAEC and EAEC: Δ*aafA* infected duodenal, ileal and colon differentiated HIE monolayers. Row one shows differentiated enteroid monolayers covered by secreted mucus droplets (denoted by the asterisk). Row two shows adherence of EAEC to mucus (yellow arrow) present on the duodenal, ileal and colon monolayers. Row three shows EAEC infected duodenal, ileal and colon monolayers but with no EAEC adherence and without the presence of mucus droplets. Row four shows EAEC: Δ*aafA* infected duodenal, ileal and colon monolayers. (D) Quantification of the EAEC aggregates in the presence and absence of mucus droplets on enteroid monolayers was quantified from average of 12 images taken at 1700x and error bars denote SEM and *** indicates significantly different (P<0.001). (E) TEM of colonoid monolayers showing vesiculation of Goblet Cell (GC) mucus and EAEC adherence to GC mucus (yellow arrow). Scale bars indicate 10µm.

Figure 5: EAEC adherence is dependent upon host intestinal mucus. (A) Experimental design explaining the chemical treatment performed to manipulate intestinal mucus and (B) showing increasing EAEC aggregative adherence to HIEMs with increased mucus production as induced by DAPT (a gamma secretase inhibitor). (C) EAEC clusters on HIEMs in the presence of N-Acetyl Cysteine (NAC), a mucolytic agent. (D-E) Quantification of EAEC aggregates in response to increasing doses of DAPT and NAC was quantified from average of 12 images taken at 100x and with field size of 2560x1920 pixel. Tukey’s multiple comparison test was performed, error bars denote SED and *** indicates significantly different (P<0.001). (F) shows representative confocal micrographs of HIEMs stained with fluorescent indicators for nuclei (DAPI, blue), MUC2 (Red), E-cadherin (Purple) and EAEC (GFP, green). HIEMs were infected with EAEC A2A:GFP that were treated with DAPT (10 µM) and NAC (3 mM). Representative of over 50 images. Scale bars indicate 10µm.

2. The Materials and Methods also lack detail, particularly the sections written in present tense. They should include the source of reagents. Including references to published protocols would be helpful. Some specific questions:

Response. We revised the methods to have more detail. The problem is always word count; reviewer 1 noted the Discussion was too long. So, it’s always a challenge to strike the balance.

Line 639 – What are 10¢ coverslips

Edited. Lines 644-645; 658-659

Line 644 – describe graded ethanol

Edited. Lines 662-663

Line 654 – clarify or reference progressively dehydrated in ethanol

Edited. Lines 674-675

Line 657- 658, please clarify “acidic mucus substances are labeled in blue, neutral mucins in purple, nuclei in red to pink and cytoplasm in pale pink”.

Edited, a more clear description about mucus and PAS/B staining has been added. Lines 678.

3. line 313 – why is 2,000 considered background?

This was performed at the Glycomics core facility at Emory. They state that for most ligands when there is no binding, the signal will harbor at around 2,000 RUs, even if there are no glycans printed on the chip. Lines 313-314

4. Lines 684-694 - A BLAST search revealed 4 mismatches at the sequence beginning to the best hit in GenBank. Please indicate the source of the gene information.

To generate the construct used to express the donor strand complemented AafA, we used the sequence of AafA provided from GenBank: AAB82330.1, which is now mentioned on Lines 709. This is the same source used by Yang et al 2011 (reference #114) and Berry et al 2014 (reference #116). Residues 11-14 are nicotinamide flucytosine dinucleotide (NFCD), but we have found additional sequences in GenBank for AafA like GenBank: AB571094.1 that have nicotinamide cytosine dinucleotide (NCD). The additional discrepancy is because we incorporated a Gly-Ser linker used to separate the his-tag and to further facilitate conformational flexible.

This information is now provided on Lines (700-726). “Constructs with this rearrangement of residues and the incorporated linker for conformational flexibility between the N and C-terminus have been shown to produce soluble and stable donor strand complemented AafA monomers (113-116). To separate the His6 tag while facilitating flexibility a short Gly-Ser linker at the N-terminus was also incorporated (atg cat cac cat cac cat cac ggt agt aat ttt tgt gat ata acg ata aca ccg gct aca aat cgt gat gtc aac gtt gac agg agc gca aat atc gac ctg agt ttt act att aga caa ccg caa cgc tgc gct gat gct ggt atg cga ata aaa gct tgg ggg gaa gcc aat cac ggt caa tta ctg ata aaa cct caa gga gga aat aaa tca gca gga ttc act ctg gcc tct cct agg ttt tct tac att ccg aat aat cca gca aac att atg aat gga ttt gtt ctt acg aat cct ggt gtt tat caa tta gga atg cag ggc tca att aca ccg gct atc ccg cta cga cca ggc cta tat gaa gta gta tta aat gct gag ctt gtg aca aat gat aac aag caa aat gca act gcg gta gca aaa act gcg acc agt act atc act gta gtg taa).”

5. Strains – please reference the source of EAEC 042: ΔAafA, E. coli: HS and describe the construction of EHEC-GFP. A table would be helpful.

Thank you. We have included a Table. Lines 1165-1168

Table 1: Details of *E. coli* strains used in the study.

| No. | Strains | Characteristics | Source |
| --- | --- | --- | --- |
| 1 | EAEC 042 | Wild-type and prototype strain | PCO |
| 2 | EAEC A2A | Clinical isolate | PCO |
| 3 | *E. coli* HS | Lab adapted commensal | PCO |
| 4 | EAEC042: ∆*aafA* | *aafA*, a major fimbrial subunit mutant | JN |
| 5 | EHEC 8624 | Wild-type strain | AT |
| 6 | ExPEC CP9 | Wild-type strain | JJ |

^#^ PCO- Dr. Pablo C. Okhuysen (UT Health, MD Anderson Cancer Institute), JN- Dr. James Nataro (University of Virginia School of Medicine), AT- Dr. Alfredo Torres (University of Texas Medical Branch), JJ- Dr. James Johnson (University of Minnesota)

Minor typos and suggestions:

1. Line 277, should be Co, et al. Corrected

2. Line 289 – symbol error. Corrected

3. Figure 2A, the second and third panels have been cut off by an extra data label.

Thank you. Revised figure has been updated.

4. Figure 2B, legend on right, the data order, listed up to down, is reversed from the left to right data order in the figure.

Thank you. This is a good suggestion but there is a good reason to keep it this way: the figures are generated based on written algorithms for hierarchical clustering and the clustering pattern would vary for each segment of intestine based on the differential expression of genes. It is automated process and hence standardization of legend in a specific order is not possible.

5. Sometimes elevated expression is red (or yellow), sometimes it is blue. It would be best to be consistent.

Revised, thank you. Figure 2

6. Figure 3A-C, For clarity, can you change “panel one” to “row 1”, etc. ?

Agreed and changed - 254-260 and Figure 3 legend

7. Figure 4. Which coronoid line or lines were used?

TC202 line was used and the details are added in Figure legend 4

8. Figure 4B. The insert needs more explanation. What cells are purple? Are the goblet cells in green? This is confusing since in the accompanying image the bacteria are stained in green. Agreed, changes have been made and updated to the Figure 4 legend.

9. Figure 6B. Please define the symbols in panel B.

A glycan key has been made and updated.

10. Figure 6C. Where is R84?

Thank you for catching this error. R84 is not involved and hence not shown in the figure.

11. Line 1097 – define FC

Abbreviated and updated.

12. Line 1167-1168. Change “bottom” to “on the right”.

Updated. Line 1145

13. Line 1171 – define NES, FDR

Defined, line 1148

14. Supporting information – please make sure all data entries are define – for example, D,J,I,C in: D.HS.over.Mock, J.HS.over.Mock, I.HS.over.Mock, C.HS.over.Mock

Key has been added in the supplementary excel sheet.
